# Airway management of a patient with coffin-lowry syndrome: a case report

**DOI:** 10.1186/s12871-024-02667-7

**Published:** 2024-08-14

**Authors:** Shaarav Ghose, Faria Nisar, Bushra Abdul Aleem

**Affiliations:** 1 Medical Student, College of Medicine, Northeast Ohio Medical University, Rootstown, OH USA; 2 Department of Anesthesiology and Pain Management, MetroHealth Medical Center, Cleveland, OH USA; 3 Northeast Ohio Medical University College of Medicine, 4209 State Route 44, 44272 Rootstown, OH USA

**Keywords:** Coffin-lowry syndrome, Anesthesia management, Intubation, Airway, Case report

## Abstract

Coffin-Lowry Syndrome (CLS) is a rare X-linked genetic disorder characterized by growth delays, facial dysmorphisms, and intellectual disabilities. Currently, there are limited published case reports regarding the anesthetic management of patients with CLS. Managing anesthesia for CLS patients can be complex due to difficult airway management. In this case report, we present a patient with CLS who underwent surgical intervention, highlighting the anesthetic considerations encountered throughout the perioperative period. We aim to summarize the difficulties involved in anesthetic management of rare conditions like CLS to improve clinical outcomes for affected individuals.

## Background

CLS is a rare X-linked genetic disease that stems from mutations in the RPS6KA3 gene on chromosome Xp22. The RPS6KA3 gene produces a protein belonging to the ribosomal S6 kinase (RSK) family. RSK proteins play important roles in the regulation of cellular differentiation and survival. Most cases of CLS occur sporadically; however, a small percentage of cases demonstrate maternal inheritance. [1] Individuals affected by this disorder are typically males demonstrating intellectual disabilities, growth delays, facial anomalies, and skeletal abnormalities. Additionally, some cases of CLS may demonstrate hearing, cardiovascular, ophthalmological, respiratory, and neurological involvement. [2] Anesthetic management of individuals with CLS can be complex, particularly related to airway management due to anatomical features like small mouth opening, retrognathia, macroglossia, and developmental delays. There are significant gaps in the literature regarding effective anesthetic strategies for CLS patients, with only a few published cases due to its rarity and the unique challenges it presents. In this case report, we aim to contribute to the limited literature on the anesthetic management of CLS patients. We share our experience in managing the anesthesia for a patient with CLS who underwent surgical intervention, highlighting the anesthetic considerations encountered throughout the perioperative period. By analyzing the difficulties involved in anesthetic management, we aim to enhance understanding of this rare genetic disorder and improve clinical outcomes for affected individuals. Informed consent for the publication of this case report was obtained from the patient’s guardians.

## Case presentation

### History and physical examination

A 17-year-old male, ASA 3, with a BMI of 16.3 kg/m2 (height:1.6 m, weight: 43 kg) and a past medical history of CLS, was scheduled for a resection of a right-sided neck mass diagnosed as a low-grade pleomorphic adenoma through fine needle aspiration cytology. Preoperative examination revealed classical features of CLS, including facial dysmorphisms: hypertelorism, thick nasal septum, prominent low-set ears, retrognathia, and macroglossia - these facial abnormalities complicated airway management due to the risk of difficult ventilation, intubation, and aspiration. The patient’s basic metabolic panel was unremarkable. Neck examination identified a 2.5 centimeter mass underneath the tail of the parotid and a smaller mass immediately below it. Subsequently, imaging findings revealed a 1.3 × 2.1 × 2.2 cm hypoechoic lesion in the subcutaneous tissues of the right neck and right jugulodigastric lymphadenopathy with the largest discrete nodes in the level IB and IIA stations, measuring up to 1.3 cm in the short axis and 2.6 cm in the greatest dimension.

**Fig. 1:**
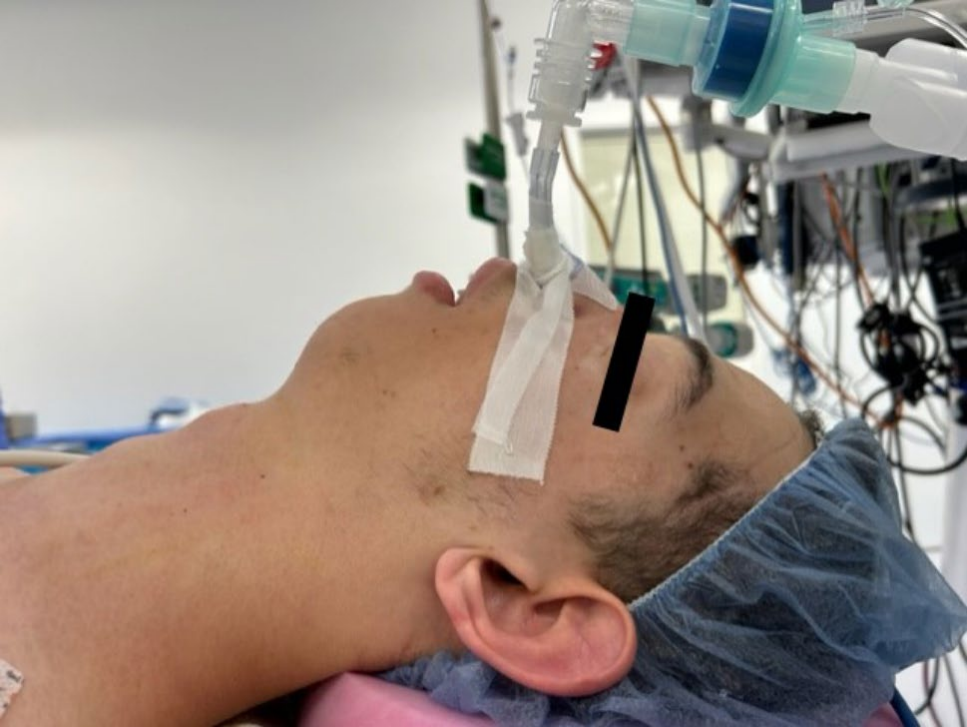
Demonstrates the patient’s facial anomalies, notably retrognathia and low-set prominent ears

Although CLS typically involves cardiovascular, skeletal, and pulmonary abnormalities, our patient had unremarkable findings in these areas. The patient did not undergo preoperative work-up such as a transthoracic echocardiogram, pulmonary function tests, or bone imaging. However, the decision was made to proceed with surgery as clinically, the patient’s cardiopulmonary status was greater than 10 Metabolic Equivalents of Task (METS). His neurological exam was significant for moderate intellectual disability, developmental delay, and autism, which made cooperation and a thorough examination - like assessment of Mallampati and mouth opening challenging. The estimated thyromental distance was less than two fingerbreadths. Given the risk of a difficult airway, we discussed our tailored anesthesia approach with the patient’s guardian, which included a high-risk, well-informed consent regarding the potential of tracheostomy as a backup plan. The patient’s neurological exam rendered the standard awake intubation approach impractical. Instead, an asleep nasal fiberoptic intubation with the primary objective of maintaining spontaneous ventilation was planned. The surgical intervention included right neck mass excision with facial nerve monitoring. Figure 1 demonstrates the various facial anomalies, notably retrognathia and low-set prominent ears.

### Intraoperative management

**Fig. 2:**
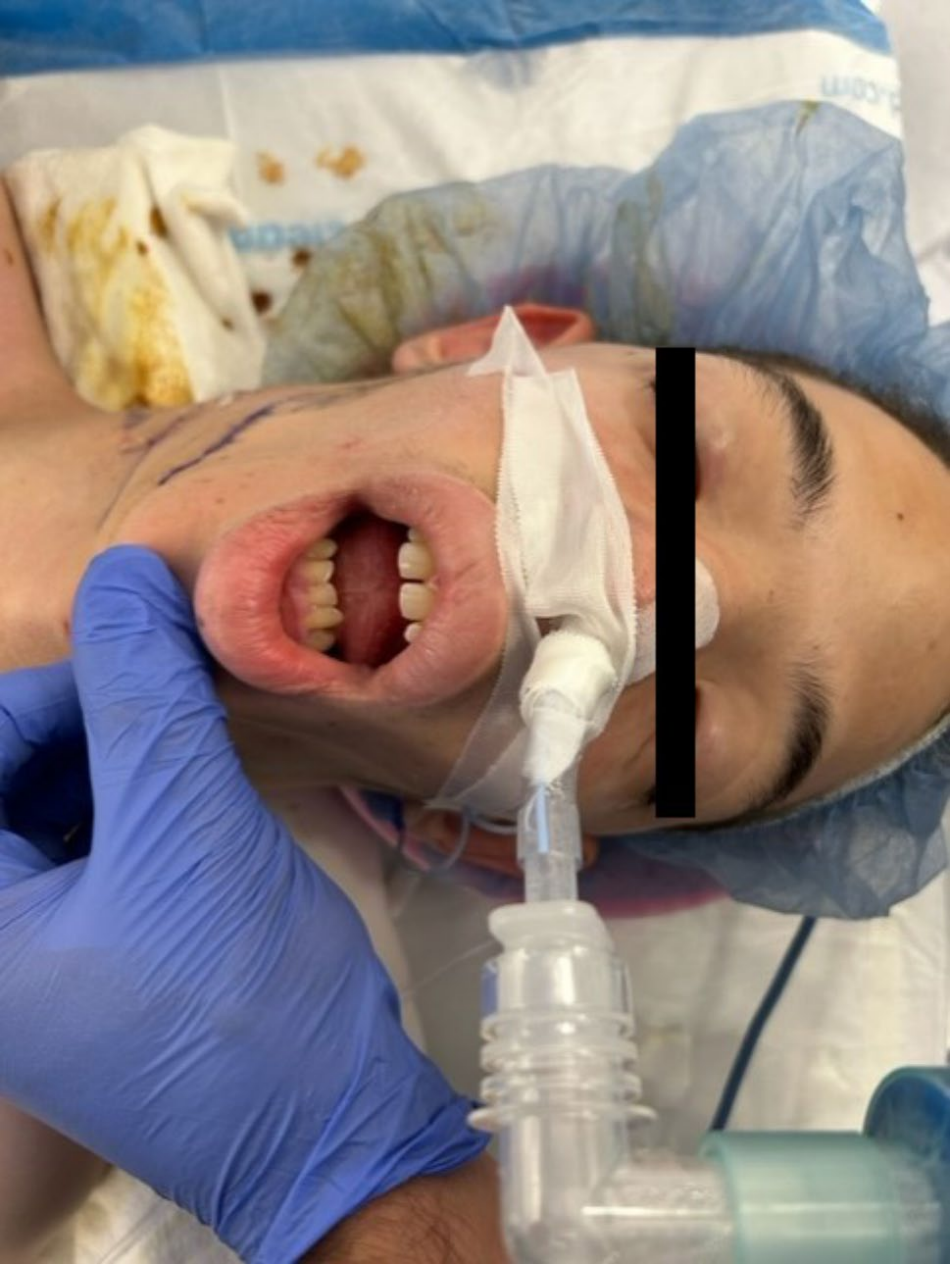
Demonstrates successful nasal intubation and facial anomalies including hypertelorism, small mouth opening, macroglossia

Our preparation encompassed several vital strategies: Firstly, we maximized patient cooperation through distraction techniques. For airway management, we prepared a range of adjuncts, including a bougie, airway exchange catheter, oral and nasopharyngeal airways, supraglottic devices, bronchoscopes, and direct and indirect laryngoscopes. Additionally, an interdisciplinary discussion with the otolaryngology team ensured preparation for a surgical airway for a failed intubation. We planned to avoid long-acting agents and discussed protocols for waking the patient if initial intubation attempts were unsuccessful. To begin the procedure, a pre-operative intravenous (IV) line was established with distraction technique, and the patient received pre-medication with IV midazolam and glycopyrrolate. The patient was then transferred to the operating room, where a combined approach of mask induction with nitrous oxide and air, along with continuous IV sedation using dexmedetomidine, was performed. Following this, sevoflurane induction was initiated with slow titration to ensure the patient maintained spontaneous ventilation. Once an adequate depth of anesthesia was achieved, nasal fiberoptic intubation with a 6.5 cuffed nasal RAE ETT was performed through the left nares as it appeared more patent and provided better access during surgery. Propofol was administered at a concentration of 10 mg/mL in divided doses, with a total dose of 50 mg given. Successful intubation was confirmed through capnometry, assessment of bilateral breath sounds, and observation of chest rise. The duration of surgery was about 2 h and 45 min. The surgical intervention proceeded without complications, and the patient tolerated the procedure well. After completion of the surgery, the patient was extubated awake and transferred to the post-anesthesia care unit (PACU) for close observation. The patient had adequate pain management and maintained oxygen saturation on room air during recovery in the PACU. Figure 2 demonstrates the successful nasal intubation with features of hypertelorism, small mouth opening, and macroglossia.

## Discussion and conclusions

CLS is a rare genetic disorder with an estimated incidence ranging from 1 in 50,000 to 100,000 individuals. CLS demonstrates an X-linked semi-dominant inheritance that leads to more severe symptoms in affected males, whereas females exhibit variable and milder effects. [3] A wide range of symptoms characterizes the syndrome; however, the most common clinical features include developmental and motor delays, facial abnormalities, and skeletal changes due to mutations in the RPS6KA3 gene. [1] Anesthetic management of CLS patients presents unique challenges that have not been extensively documented in the literature, with currently only a few published case reports available. The various strategies in anesthetic management for these specific cases are summarized in Table 1. Our case report discusses why we decided to opt against the standard awake intubation approach for our patient with CLS and instead proceed with asleep nasal fiberoptic intubation while maintaining spontaneous ventilation. We aim to provide novel insights by addressing the unique challenges we experienced in the anesthetic management of this patient.

**Table 1:** Summary of the anesthetic management of previously reported Coffin-Lowry syndrome cases

Case Report	Patient Information	Procedure	Anesthesia management
Hashiguchi et al. [5]	33-year-old female	Cervical cyst resection	Slow induction with propofol and easy mask ventilation. Administration of vecuronium, midazolam, and fentanyl. Direct laryngoscopy with Macintosh laryngoscope.
Singh PM et al. [6]	14-year-old male	Vitreoretinal surgery	Gradual induction with sevoflurane and oxygen with mask ventilation. Administration of fentanyl and atracurium. Placement of Proseal laryngeal mask airway (LMA) and LMA was maintained throughout surgery.
Kawana et al. [7]	12-year-old male	Posterior Cervical spinal fusion	Slow induction with sevoflurane and oxygen. LMA Fastrach was inserted and fiberoptic-guided intubation through the LMA Fastrach with rocuronium.
Hirakawa et al. [8]	24-year-old male	Laminectomy	Sedation with lidocaine and propofol. Fiberoptic-guided intubation with spontaneous breathing maintained. Maintenance of anesthesia was achieved with propofol and remifentanil.

When encountering challenging intubations, awake fiberoptic intubation is often preferred, and studies have demonstrated a success rate of over 90% in managing these patients. [4] Given our patient’s presentation, we concluded that these findings made the standard approach impractical for our case. Firstly, the patient’s developmental delay impedes their ability to cooperate during awake intubation. These cognitive challenges make it difficult to ensure patient cooperation during the procedure, which could lead to distress and resistance, thus compromising safety. We addressed this communication barrier using constant distraction and redirection techniques. Secondly, the facial dysmorphisms exhibited by the patient, such as retrognathia and macroglossia, present anatomical challenges, making traditional mask ventilation and intubation less feasible. Given these expected challenges, we decided on an asleep nasal fiberoptic intubation while ensuring spontaneous ventilation.

The nasal fiberoptic intubation technique allows for a more controlled and gradual approach to securing the airway, thus reducing the risk of trauma to structures or failure to secure the airway. Additionally, the patient’s sedation ensured optimal comfort and alleviated any potential anxiety or resistance related to the procedure. Maintaining spontaneous ventilation in the patient was crucial to preserve protective airway reflexes and minimize the risk of respiratory complications during the procedure. This case report demonstrates the complexities involved in the anesthetic management of individuals with rare genetic disorders such as CLS. We emphasize the necessity of customizing the airway management to optimize patient safety. In this case, we conducted a tailored approach that combined pre-medication, sedation, and asleep nasal fiberoptic intubation while ensuring the patient maintained spontaneous ventilation. To optimize cooperation and reduce anxiety, we also used distraction techniques due to the patient’s developmental delays. This approach allowed for a controlled induction and enabled us to secure the airway effectively while minimizing risk and maximizing patient comfort.

In conclusion, using fiberoptic intubation while maintaining spontaneous breathing is a preferred approach for CLS patients compared to awake intubation due to the challenges associated with cognitive disabilities, airway anomalies, the risk of aspiration, and overall patient comfort. Our case report adds novel information by highlighting the use of distraction techniques to enhance cooperation in a patient with significant developmental delays. Additionally, we detail the specific preparations undertaken by our team to provide guidance on how to prepare for patients with similar conditions. Further research is needed to develop specific guidelines for the anesthetic management of individuals with CLS and other rare genetic disorders. By sharing our experiences, this case report aims to guide anesthetic management strategies for affected individuals to improve overall clinical outcomes.

## Data Availability

No datasets were generated or analysed during the current study.

## Declarations

### Clinical trial registration

This manuscript does not include a clinical trial; instead, it provides a summarized table of previously published case reports.

## Abbreviations

CLS: Coffin-Lowry Syndrome
RSK: ribosomal S6 kinase
HIPAA: Health Insurance Portability and Accountability Act
PACU: post-anesthesia care unit
LMA: laryngeal mask airway
IV: intravenous
EET: endotracheal tube
METS: Metabolic Equivalents of Task
ASA: American society of Anesthesiologists
